# Exosome‐derived long non‐coding RNA ADAMTS9‐AS2 suppresses progression of oral submucous fibrosis via AKT signalling pathway

**DOI:** 10.1111/jcmm.16219

**Published:** 2020-12-20

**Authors:** Shanghui Zhou, Yun Zhu, Zhenming Li, Yonggan Zhu, Zhijing He, Chenping Zhang

**Affiliations:** ^1^ Department of Oral & Maxillofacial—Head & Neck Oncology Shanghai Ninth People's Hospital College of Stomatology Shanghai Jiao Tong University School of Medicine Shanghai China; ^2^ National Clinical Research Center for Oral Diseases Shanghai China; ^3^ Shanghai Key Laboratory of Stomatology & Shanghai Research Institute of Stomatology Shanghai China; ^4^ Department of Nursing Shanghai Ninth People's Hospital Shanghai Jiao Tong University School of Medicine Shanghai China; ^5^ Department of Oral and Maxillofacial Surgery The Second Xiangya Hospital Central South University Changsha China

**Keywords:** ADAMTS9‐AS2, EMT, metastasis, OSCC, OSF

## Abstract

Oral submucosal fibrosis (OSF) is one of the pre‐cancerous lesions of oral squamous cell carcinoma (OSCC). Its malignant rate is increasing, but the mechanism of malignancy is not clear. We previously have elucidated the long non‐coding RNA (lncRNA) expression profile during OSF progression at the genome‐wide level. However, the role of lncRNA *ADAMTS9‐AS2* in OSF progression via extracellular communication remains unclear. lncRNA *ADAMTS9‐AS2* is down‐regulated in OSCC tissues compared with OSF and normal mucous tissues. Low *ADAMTS9‐AS2* expression is associated with poor overall survival. *ADAMTS9‐AS2* is frequently methylated in OSCC tissues, but not in normal oral mucous and OSF tissues, suggesting tumour‐specific methylation. Functional studies reveal that exosomal ADAMTS9‐AS2 suppresses OSCC cell growth, migration and invasion in vitro. Mechanistically, exosomal ADAMTS9‐AS2 inhibits AKT signalling pathway and regulates epithelial‐mesenchymal transition markers. Through profiling miRNA expression profile regulated by exosomal ADAMTS9‐AS2, significantly enriched pathways include metabolic pathway, PI3K‐Akt signalling pathway and pathways in cancer, indicating that exosomal ADAMTS9‐AS2 exerts its functions through interacting with miRNAs during OSF progression. Thus, our findings highlight the crucial role of ADAMTS9‐AS2 in the cell microenvironment during OSF carcinogenesis, which is expected to become a marker for early diagnosis of OSCC.

## 1 INTRODUCTION

Oral squamous cell carcinoma (OSCC) is one of the most common types of head and neck malignancies, which is characterized by rapid progression, extensive infiltration, easy neck lymphatic metastasis and poor prognosis.^1^ OSCC evolves from precancerous lesions, including oral submucous fibrosis (OSF), oral leucoplakia (OLK), oral lichen planus (OLP) and erythroplakia.^2^ OSF has its distinct geographical pathogenesis, and the first case was reported in 1985 in mainland China.^3^ The pathology of OSF can be divided into the early, middle and advanced stages, which are possible processes to OSF carcinogenesis. Recent studies have shown that the cancerous rate of OSF is increasing by reaching 3%‐19%.^4^, ^5^ Thus, identification of key molecular events in the malignant progression of OSF will help improve the early diagnosis and prevention of OSCC.

Exosomes are extracellular messengers that transport and exchange substances between tumour cells and microenvironment.^6^, ^7^ Long non‐coding RNA (lncRNAs) can be packaged into exosomes and act as messengers for intercellular communication, participating in the regulation of cell microenvironment. Deregulation of exosomal lncRNA affects the tumour microenvironment such as angiogenesis, metastasis and drug resistance and thus contributes to tumorigenesis.^8^ Our preliminary work has interpreted the lncRNA expression profile during the malignant evolution of normal oral mucosa‐OSF‐OSCC at the genome‐wide level for the first time.^9^ We found that the key signalling pathways involved in OSF carcinogenesis are chemokine and cytokine‐mediated inflammatory signalling pathway, Wnt signalling pathway and angiogenesis signalling pathway, which are closely related to alterations in the tumour microenvironment.

The ADAMTS (a disintegrin and metalloproteinase with thrombospondin motifs) family promotes or inhibits the tumorigenic potential of tumour cells through alterations in tumour microenvironment.^10^ ADAMTS family proteins are involved in a variety of biological processes, including fibrosis, angiogenesis, cell invasion and metastasis, and tumorigenesis.^11^ The role of ADAMTS family proteins in fibrosis of the myocardium,^12^, ^13^ liver^13^, ^14^, ^15^ and lung^13^, ^16^, ^17^ has been well studied. Moreover, genetic and epigenetic alterations (mutation, CpG methylation) of ADAMTS family proteins in multiple tumours including head and neck cancer indicate their direct roles to cancer initiation and progression through exerting oncogenic or anti‐tumour effects.^18^ ADAMTS family genes have been identified to be involved in the development and metastasis of head and neck cancer. *ADAMTS14* gene polymorphisms as a risk factor for oral cancer together with environmental carcinogens (betel nut chewing and smoking) contribute to oral cancer initiation.^19^ *ADAMTS9* is down‐regulated by promoter CpG methylation in nasopharyngeal carcinoma and inhibits angiogenesis through regulating the tumour microenvironment.^20^ However, the regulatory mechanism of the ADAMTS family on OSF malignancy, a disease of oral mucosal fibrosis, remains unknown.

In this study, we explored the role of ADAMTS family members in OSF carcinogenesis and found significant differences in the expression of lncRNA ADAMTS9‐AS2. LncRNA ADAMTS9‐AS2 was highly expressed in normal oral mucosal tissues but down‐regulated in OSF and OSCC tissues, which is associated with poor prognosis. We found that exosomes carrying ADAMTS9‐AS2 inhibit cell proliferation and metastasis of OSCC cells. Mechanistically, ADAMTS9‐AS2 suppresses PI3K‐AKT signalling pathway and epithelial‐mesenchymal transition (EMT). Our findings indicate that exosome‐derived ADAMTS9‐AS2 suppresses the progression of oral submucous fibrosis.

## 2 MATERIALS AND METHODS

### 2.1 Cell lines, tumour samples and normal tissues

Two OSCC cell lines used in this study included CAL‐27 and SCC‐9, purchased from the American Type Culture Collection (ATCC). Primary fibroblast cultures of human buccal mucosa were grown and maintained according to the procedures described.^21^, ^22^ Biopsy specimens were derived from histologically normal oral mucosa and OSF patient. OSF cells between the fifth and seventh passages were collected. Cells were cultured in Dulbecco's modified Eagle's medium (DMEM; Gibco) or DMEM/F12 (1:1) medium (Gibco‐BRL) supplemented with 10% foetal bovine serum (Gibco‐BRL), 1% glutamine and 1% penicillin‐streptomycin, at 37°C in a humidified atmosphere containing 5% CO_2_.

Oral squamous cell carcinoma, OSF and normal oral mucosa tissues were obtained from patients under surgical resection at the Second Xiangya Hospital and Xiangya Hospital, Central South University (Changsha, China), and Shanghai Ninth People's Hospital, Shanghai Jiaotong University School of Medicine (Shanghai, China), from January 2016 to June 2017, as described previously.^9^ The patients' informed consent had been obtained under a protocol reviewed and approved by the Institutional Review Boards of the Xiangya School of Medicine or Shanghai Jiaotong University School of Medicine. All samples were pathologically confirmed by two pathologists independently. Archived DNA from independent cohorts includes normal mucous samples (n = 10), OSF samples (n = 10) and OSCC samples (n = 20).^23^, ^24^

### 2.2 Reverse transcription (RT) and quantitative real‐time PCR

Total RNA was extracted using TRIzol reagent (TaKaRa) and reverse‐transcribed with a Reverse Transcription System (Promega). qRT‐PCRs were performed with an SYBR Green PCR Master Mix Kit (Invitrogen) on the ABI StepOne Real‐Time PCR System (Applied Biosystems). The relative expression of ADAMTS9‐AS2 was estimated using the threshold cycle (Ct) method, and all assays were performed in triplicate. GAPDH was used as a loading control. The primer sequences used in this study were listed as follows: ADAMTS9‐AS2: 5′‐CTTTAAGACCCACGAACGAC‐3′ and 5′‐TACTTGAGGAGAAAGCGAAA‐3′; and GAPDH: 5′‐TGACTTCAACAGCGACACCCA‐3′ and 5′‐CACCCTGTTGCTGTAGCCAAA‐3′.

### 2.3 Lentiviral transduction and screening of stable strains

LncRNA ADAMTS9‐AS2 lentiviral expression vector was constructed by Gikai Biotechnology Co., Ltd. Lentiviral transduction was performed following the manufacturer's instructions. GFP expression was observed under a fluorescence microscope with a fluorescence rate of about 80% and cell confluence of about 80% for the lentiviral infection. Seventy two hours after infection, cells in good growth status with an infection efficiency of about 80% were screened with antibiotics for establishing stably expressing cell lines.

### 2.4 Exosome isolation and purification

Exosome isolation and purification were performed using ExoQuick™ Exosome Precipitation Solution (SBI) according to the manufacturer's protocol. When cell cultures reached 90% confluence, cells were washed with PBS and incubated with fresh culture medium without FBS or penicillin‐streptomycin for 48‐72 hours, and the conditioned medium was collected. Briefly, cells and cell debris were removed by centrifuging at 3000×*g*. The appropriate volume of ExoQuick Exosome Precipitation Solution (ExoQuick, SBI) was added to transferred supernatant and remained upright overnight at +4°C. ExoQuick mixture was centrifuged at 1500×*g* for 30 minutes. After centrifugation, the exosome pellet was resuspended in 100 µL 1X PBS for RNA/protein extraction or cell treatment.

For transmission electron microscopy, exosomes were placed on a copper grid. 5‐10 μL of exocrine suspension was aspirated gently onto the front of the copper‐loaded mesh and carefully dried with clean filter paper after 1 minute. 10‐20 μL of EM solvent was added in drops and carefully vacuumed off with a clean filter paper, and transmission electron microscopy was performed the next day. The exosomes then were quantified using NanoSight NS300 (Malvern Instruments Ltd.).

### 2.5 Bisulphite treatment and promoter methylation analysis

Methylation‐specific PCR (MSP) was performed as described previously.^23^, ^24^ MSP was conducted for 40 cycles at the annealing temperatures of 60°C for M and 58°C for U *ADAMTS9‐AS2*m1: 5′‐GATAGCGTATTTCGGGAGTTAC‐3′; *ADAMTS9‐AS2*m2: 5′‐TCTACCTTTCAATAAAAAAATCGAA‐3′; *ADAMTS9‐AS2*u1: 5′‐GATAGTGTATTTTGGGAGTTATGG‐3′; and *ADAMTS9‐AS2*u2: 5′‐TCTACCTTTCAATAAAAAAATCAAA‐3′.

### 2.6 Cell proliferation assay

Cell proliferation assay was performed using CellTiter 96^®^ AQ_ueous_ One Solution Cell Proliferation Assay (MTS) (Promega) according to the manufacturer's instructions. Cells (2000 cells/well) were seeded in 96‐well plates and cultured for days 1, 2, 3, 4 and 5. 20 μL of MTS was added to each well and incubated for 4 hours each day, and then, the absorbance value (OD) was detected at 490 nm. All experiments were repeated three times.

### 2.7 Colony formation assay

Stably transfected CAL‐27 and SCC‐9 cells were plated in six‐well plates (400‐1000 cells/well) with three replicate wells per experimental group. The inoculated cells were continued in the incubator for 14 days or until the number of cells in the majority of individual clones was greater than 50, with culture medium changes every 3 days and cell status observed. Cell clones were photographed under a fluorescence microscope. 4% paraformaldehyde was added per well, cells were fixed and PBS washed, the crystalline violet staining solution was added per well, and cells were stained for 10‐20 minutes. Pictures were taken with a phase‐contrast microscope, and clones were counted.

### 2.8 Transwell migration and invasion assays

In vitro Transwell^®^ assays were performed using 24‐well Transwell chambers (8‐μm pore size, Corning) with or without Matrigel for cell migration and invasion, respectively. Cells were collected, resuspended in serum‐free medium and added to the upper chamber (10^5^ cells), and 30% FBS medium to the lower chamber. Cells were incubated at 37°C for 48 hours. The chambers were inverted on absorbent paper to remove the medium, the non‐transformed cells were gently removed from the chambers with a cotton swab, and the chambers were placed in 4% paraformaldehyde fixative for 30 minutes and were stained for Giemsa (Sigma‐Aldrich). Migrated cells were photographed with a random selection of the field of view using a phase‐contrast microscope. The number of metastatic cells per field for each group (migratory cells per field) was counted. All experiments were independently repeated three times.

### 2.9 Western blot analysis

Western blotting was performed as previously described.^25^, ^26^ Primary antibodies used in this study are as follows: AKT‐total (#4691), phospho‐AKT(Ser473) (#4060), E‐cadherin (#4065) N‐cadherin (#13116) and GAPDH (#2118) (Cell Signaling); vimentin (V6630; Sigma‐Aldrich); and antimouse IgG‐HRP (P0161) and anti‐rabbit IgG‐HRP (P0448) (Dako). Immunoreactive bands were visualized using Western Blot Luminol Reagent (GE Healthcare Bio‐Sciences) according to the manufacturer's protocol. The signals were visualized using a ChemiDoc™ Imaging System (Bio‐Rad).

### 2.10 Statistical analysis

Data were presented as mean ± standard error of the mean (SEM). Differences between the expression levels of lncRNAs in normal mucous, OSF and OSCC tissues were evaluated by one‐way or two‐way analysis of variance (ANOVA). The Kaplan‐Meier survival analysis was used to evaluate the correlation of ADAMTS9‐AS2 expression level with patient survival outcome. Statistical analysis was performed in excel or using SPSS 21.0 package. *P* < .05 was considered significant.

## 3 RESULTS

### 3.1 Down‐regulated ADAMTS9‐AS2 is associated with poor prognosis of OSCC patients

**FIGURE 1:**
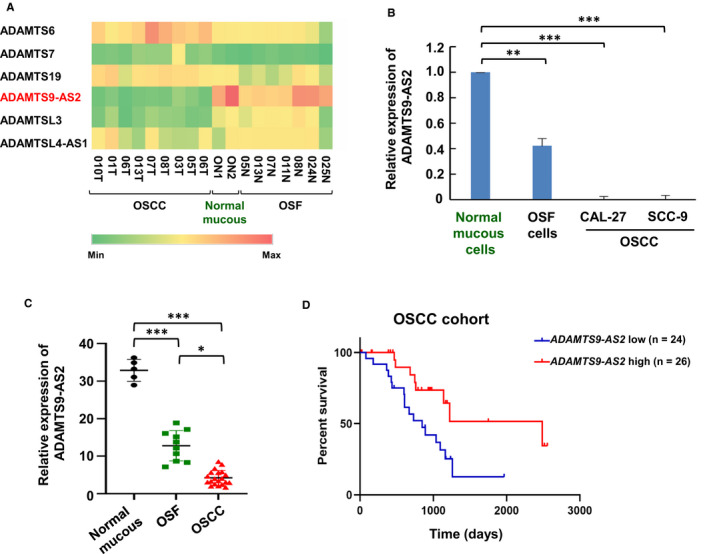
LncRNA ADAMTS9‐AS2 is down‐regulated during OSF carcinogenesis and associated with poor prognosis in OSCC patients. A, Heat map of ADAMTS9‐AS2 family members differentially expressed in normal mucous, OSF and OSCC samples. B, ADAMTS9‐AS2 RNA levels were quantified by qRT‐PCR in normal mucous, OSF and OSCC cells. **P < .01; ***P < .0001. C, qRT‐PCR analysis of ADAMTS9‐AS2 in tissue samples from normal mucous (n = 5), OSF (n = 10) and OSCC (n = 19) samples. ADAMTS9‐AS2 expression was normalized to GAPDH (‐ΔCt). Data were presented as 2^‐ΔΔCt^. *P < .05; ***P < .0001. D, Kaplan‐Meier curves for overall survival of OSCC patients with low vs high expression of ADAMTS9‐AS2

Using next‐generation sequencing (NGS), we previously identified the lncRNA landscape during OSF malignant progression in 2 normal mucous tissues, 8 OSF tissues with different stages and 8 OSCC combined with OSF tissues (Gene Expression Omnibus ID GSE106534). To identify the involvement of ADAMTS family members during OSF progression, 6 ADAMTS family genes were selected with rigorous criteria including fold change >4 and transcript abundance >100. Then, we chose the significantly differentially expressed transcript during OSF carcinogenesis, lncRNA ADAMTS9‐AS2 as a target, which has the highest expression in normal mucous tissues, moderate expression in OSF tissues and lowest expression in OSCC combined with OSF tissues (Figure 1A). Quantitative real‐time PCR (qRT‐PCR) confirmed *ADAMTS9‐AS2* was highly expressed in normal oral epithelium cells compared with OSF cells and OSCC cells. Moreover, significantly higher *ADAMTS9‐AS2* expression was also found in OSF cells than that in CAL‐27 and SCC‐9 cells (**P < .01; ***P < .001) (Figure 1B).

We next examined *ADAMTS9‐AS2* expression in normal mucous, OSF and OSCC samples by qRT‐PCR analysis. *ADAMTS9‐AS2* expression was found to be down‐regulated in OSCC tissues compared with OSF and normal mucous tissues (*P < .05; ***P < .001) (Figure 1C). Survival analysis showed that low *ADAMTS9‐AS2* expression was associated with poor overall survival (OS; *P* < .01) (Figure 1D). Thus, these data suggested that *ADAMTS9‐AS2* plays an important role in OSF progression.

### 3.2 *ADAMTS9‐AS2* methylation mediates its reduction in OSF tumorigenesis

**FIGURE 2:**
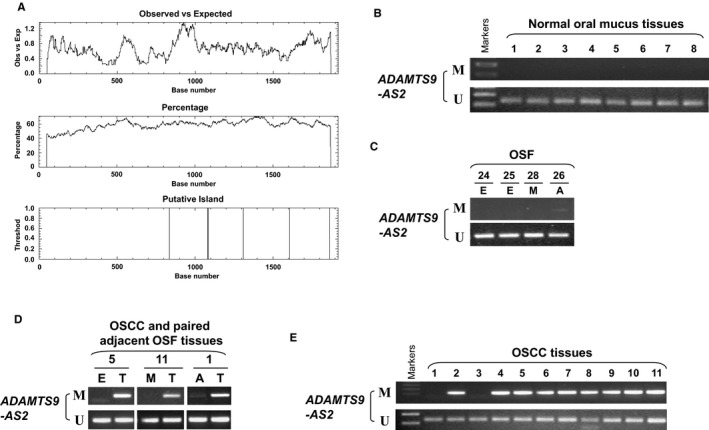
*ADAMTS9‐AS2* methylation during OSF progression. (A) Promoter CpG island analysis of the *ADAMTS9‐AS2* promoter and exon 1 regions by the EMBOSS Cpgplot (http://www.ebi.ac.uk/Tools/seqstats/emboss_cpgplot/). Representative *ADAMTS9‐AS2* methylation by MSP in (B) normal oral mucosa tissues, (C) OSF tissues, (D) OSCC and paired adjacent OSF tissues, and (E) OSCC tissues. M, methylated; U, unmethylated; E, early stage of OSF; M, moderately advanced stage of OSF; A, advanced stage of OSF; N, normal tissue; T, OSCC

We next assessed the possible regulatory mechanism of *ADAMTS9‐AS2* reduction in OSF tumorigenesis. We firstly examined the presence of CpG island (CGI) in the *ADAMTS9‐AS2* promoter and exon 1 by bioinformatics analysis. The region spanning the *ADAMTS9‐AS2* promoter and exon 1 fulfilled the criteria of a CpG island: GC content >50% and observed/expected CpG ratio >0.60 in a 1920‐bp region (Figure 2A). We thus examined promoter methylation of *ADAMTS9‐AS2* in normal oral mucous tissues and OSF tissues with different stages. Rare methylation of *ADAMTS9‐AS2* was detected in 10 normal oral tissues and 10 OSF tissues from the early stage and moderately advanced stage, and weak methylation was found in 4 OSF tissues with advanced stage (Figure 2B,C). We further examined its methylation in OSCC and their paired adjacent OSF *ADAMTS9‐AS2* was frequently methylated in 15 of 20 (15/20, 75%) OSCC tissues, but not in their paired adjacent OSF tissues (Figure 2D,E). These data suggest that *ADAMTS9‐AS2* methylation is a tumour‐specific event in the carcinogenesis of OSF.

### 3.3 Exosomal ADAMTS9‐AS2 serves as a mediator in intercellular communication

**FIGURE 3:**
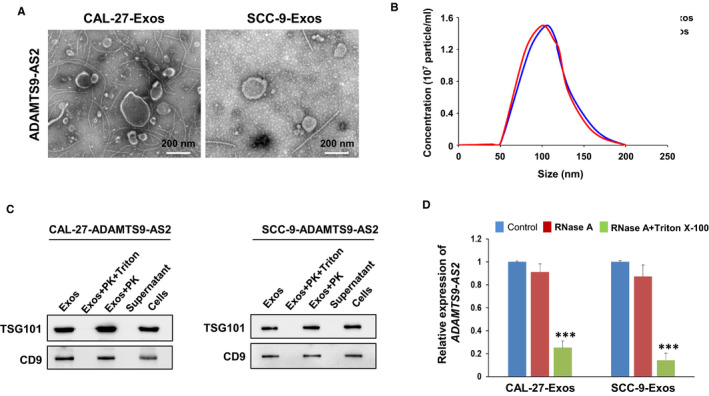
Identification and analysis of exosomal ADAMTS9‐AS2 derived from OSCC cells. A, Exosomes isolated from supernatants of ADAMTS9‐AS2–expressing OSCC cells were analysed by transmission electron microscopy. Scale bar = 200 nm. B, Size distributions and numbers of exosomes isolated from the culture media of ADAMTS9‐AS2–expressing OSCC cells by NanoSight particle tracking. C, Western blot analysis of the representative proteins as positive markers of exosomes (TSG101, CD9) in exosome extracts, with or without treatment of Triton X‐100 + proteinase K (PK) or proteinase K (PK) alone. D, qRT‐PCR analysis of ADAMTS9‐AS2 in the CM of OSCC cells with treatment of RNase (2 mg/mL) alone or combined with Triton X‐100 (0.1%) for 20 min (n = 3). Exos, exosomes

We further isolated and identified the exosomes from the culture medium of OSCC cell lines (CAL‐27 and SCC‐9) with ADAMTS9‐AS2 expression. We examined exosomes in ADAMTS9‐AS2–expressing OSCC cells by transmission electron microscopy (TEM) and found the presence of typical circle‐shaped morphologies (Figure 3A). The nanoparticle tracking analysis (NTA) showed the size of exosomes was about 100‐150 nm, with distribution ranged from 50 to 200 nm (Figure 3B).

As exosomes are small vesicles (30‐150 nm) within the lipid membrane, and composed of protein and/or RNA cargo, we next used proteinase K and/or Triton X‐100 treatment to determine whether derived ADAMTS9‐AS2 exosomes were protected by the exosomal membrane. We examined two exosomal protein markers TSG101 (vesicular trafficking protein) and CD9 (tetraspanin) in the particles isolated from the culture medium (CM) of ADAMTS9‐AS2–expressing OSCC cells by Western blot to verify the presence of exosomes in our study. Results revealed that TSG101 and CD9 proteins were clearly expressed in exosomes extracts with proteinase K treatment alone and cell lysates, but not expressed in the supernatant and exosomes extracts with both proteinase K and Triton X‐100 treatments that membranes have been permeabilized (Figure 3C). We further investigated whether the existing pattern of extracellular ADAMTS9‐AS2 in exosomes was protected from RNase A degradation. We found that RNase treatment did not change the expression levels of ADAMTS9‐AS2 in exosomes too much, but its expression was significantly reduced when exosomes were treated with both RNase and Triton X‐100 (***P < .001) (Figure 3D). These data indicate that extracellular ADAMTS9‐AS2 was mainly wrapped by the membrane and packaged in exosomes instead of being directly released.

### 3.4 Exosomal ADAMTS9‐AS2 inhibits OSCC tumour cell growth

**FIGURE 4:**
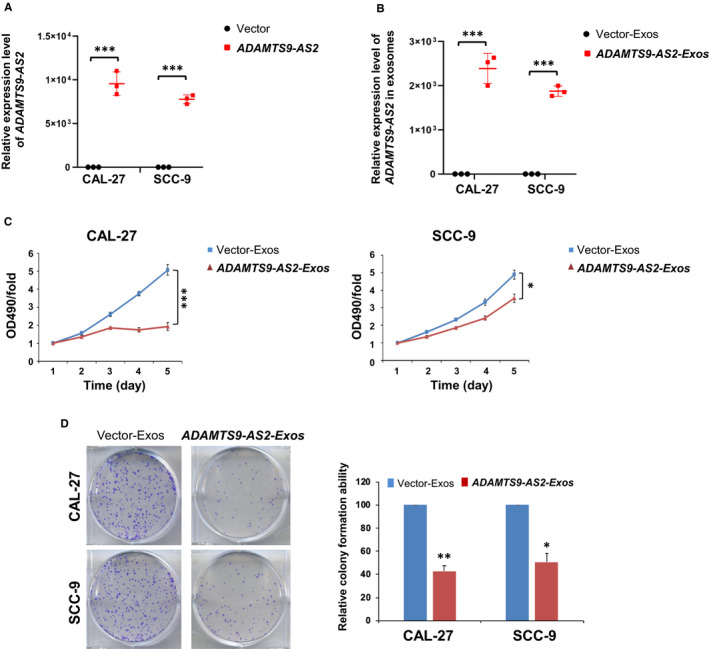
Exosomal ADAMTS9‐AS2 inhibits OSCC cell proliferation. ADAMTS9‐AS2 expression in (A) ADAMTS9‐AS2–overexpressing OSCC cells and (B) their corresponding exosomes by qRT‐PCR analysis. ***P < .001. C, Cell viabilities were evaluated after incubated with exosomes (10 μg/mL) in CAL‐27 and SCC‐9 cells by MTT. *P < .05; ***P < .001. D, Colony formation of OSCC cells with or without treatment of ADAMTS9‐AS2–expressing exosomes (left panel). Rates are shown as mean ± SD (right panel) from three independent experiments. *P < .05; **P < .01. Exos, exosomes

We next examined ADAMTS9‐AS2 expression in exosomes isolated from the culture medium of OSCC cell lines (CAL‐27 and SCC‐9) by real‐time PCR. ADAMTS9‐AS2 overexpression via lentiviral infection of ADAMTS9‐AS2 plasmid led to an obvious increase of ADAMTS9‐AS2 levels in both the OSCC cells and their secreted exosomes (***P < .001) (Figure 4A,B), suggesting that ADAMTS9‐AS2 mainly exists in exosome form. We further investigated the effect of ADAMTS9‐AS2 on cell growth in vitro. The cell viability and colony formation assays in OSCC cells incubated with OSCC cell‐secreted exosomes were analysed. The exosomes secreted from OSCC cells with ADAMTS9‐AS2 expression significantly inhibited OSCC cell viability (**P < .01; ***P < .001) (Figure 4C). Moreover, the exosomes secreted by ADAMTS9‐AS2–overexpressing cells (ADAMTS9‐AS2‐Exos) strongly suppressed the growth of both CAL‐27 and SCC‐9 cells to 40%‐50% (*P < .05; **P < .01) by colony formation assay (Figure 4D). These data suggest that exosomal ADAMTS9‐AS2 inhibits tumour cell growth during oral tumorigenesis.

### 3.5 Exosomal ADAMTS9‐AS2 suppresses OSCC tumour cell metastasis, EMT and AKT signalling pathway

**FIGURE 5:**
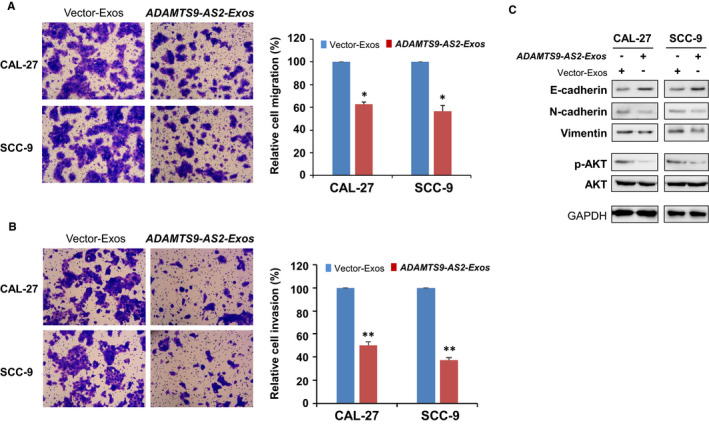
Exosomal ADAMTS9‐AS2 suppressed OSCC cell migration and invasion. Cell migration (A) and invasion (B) abilities of OSCC cells upon treatment with ADAMTS9‐AS2–expressing exosomes were measured by Transwell assays. Representative images were photographed following fixation and staining. Scale bars: 50 μm. Data are presented as the mean ± SD. *P < .05; **P < .01. C, Western blot analysis of EMT markers and AKT signalling pathway in OSCC cells with treatment of ADAMTS9‐AS2–expressing exosomes and control exosomes. Exos, exosomes

As some ADAMTS family members link with tumorigenesis via regulating cell migration and cell signalling pathways, the role of exosomal ADAMTS9‐AS2 in OSCC cell migration and invasion abilities was further analysed by Transwell^®^ assays. Results showed that ADAMTS9‐AS2‐Exos significantly inhibited the migratory and invasive abilities of CAL‐27 and SCC‐9 cells (*P < .05; **P < .01) (Figure 5A,B). We next examined the changes of the AKT signalling pathway and EMT markers in OSCC cells. We found exosomal ADAMTS9‐AS2 up‐regulated expression level of epithelial marker E‐cadherin and reduced the expression levels of mesenchymal markers N‐cadherin and vimentin in CAL‐27 and SCC‐9 cells (Figure 5C).

To identify cell signalling pathways deregulated by exosomal ADAMTS9‐AS2 during OSCC tumorigenesis, we thus examined the AKT signalling pathway by Western blot. Results showed that obvious down‐regulation of phosphorylated AKT in CAL‐27 and SCC‐9 cells with incubation of exosomal ADAMTS9‐AS2 is consistent with other studies (Figure 5C). These data suggest that exosomal ADAMTS9‐AS2 suppresses tumour metastasis of OSCC, which may be through regulating EMT and AKT signalling pathways.

### 3.6 miRNA expression profile of exosomal ADAMTS9‐AS2 in OSCC cells

**FIGURE 6:**
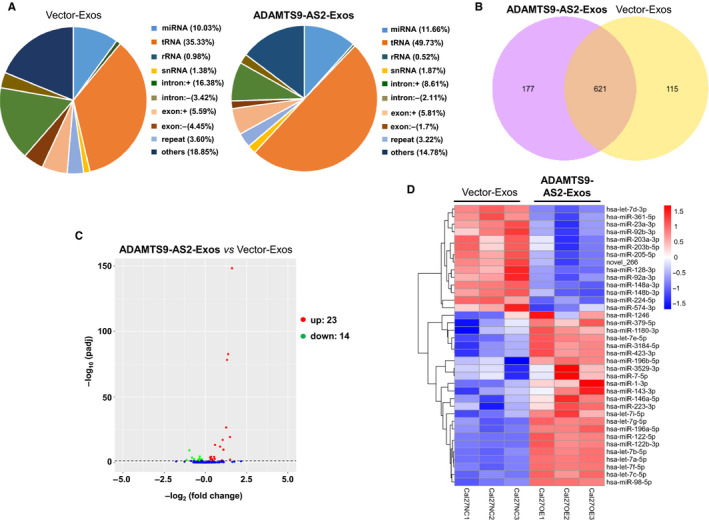
miRNA expression profiles of exosomal ADAMTS9‐AS2. A, The percentage of small RNA categories in all reads mapped to non‐coding RNA databases. B, Venn diagram showing the unique and overlapping miRNAs between ADAMTS9‐AS2–expressing exosome‐treated groups and control groups. C, Volcano plot showing differential miRNAs in OSCC cells treated with ADAMTS9‐AS2–expressing exosomes and control exosomes. *P* < .01 and fold change >2 were considered significant. D, Heat map diagram of differential miRNA expression by exosomal ADAMTS9‐AS2. Expression values shown are mean‐centred. Red, increased expression; blue, decreased expression; and white, mean value. Exos, exosomes

Exosomes contain both mRNA and microRNA, which are involved in cell communication and exert multiple biological functions. We thus further investigated the miRNA expression profile of exosomes derived from CAL‐27 cells with ADAMTS9‐AS2 overexpression using Illumina HiSeq 2500 high‐throughput sequencing (miRNA‐seq). Initially, each sample got 12 448 626 ~ 8 094 643 total reads (Table S1). After removing low‐quality reads, contaminants and adaptors, each sample clean reads were screened by small RNAs (sRNAs) within a certain length range for subsequent analysis. Because of the specificity of exosomal samples, which contain a large number of degradation fragments of other RNAs, high levels of reads fragments were concentrated at 30‐32 nucleotides (nt). Length‐screened sRNAs were mapped to the non‐coding (nc) RNA databases. The reads identified for categories of small RNA (miRNA, rRNA, tRNA, snRNA, snoRNA, piRNA and Y_RNA) and unannotated RNAs (others) (Figure 6A). The percentage of miRNAs in the total RNA isolated ADAMTS9‐AS2 exosomes corresponded to 11.66%.

To identify the conserved miRNAs, all ncRNA reads from exosome libraries were compared with the specified range sequence in miRBase. A total of 736 and 798 types of known miRNAs in control and ADAMTS9‐AS2 exosomes were identified, with 621 miRNAs that were simultaneously identified in both groups (Figure 6B, Table S2). We next analysed differentially expressed miRNAs in ADMTS9‐AS2 exosomes using a twofold change and corrected level of significance (*P* adj < .05, q value < 0.01) as the threshold cut‐off. We found that 37 miRNAs were significantly different between control and ADAMTS9‐AS2 exosomes, including 23 up‐regulated miRNAs and 14 down‐regulated miRNAs, as shown by the Venn diagram and hierarchical clustering (Figure 6C,D, Table S3). These results indicate the involvement of differentially expressed miRNAs in ADAMTS9‐AS2–induced OSCC pathogenesis.

### 3.7 Functional enrichment of target genes of miRNAs regulated by exosomal ADAMTS9‐AS2

**FIGURE 7:**
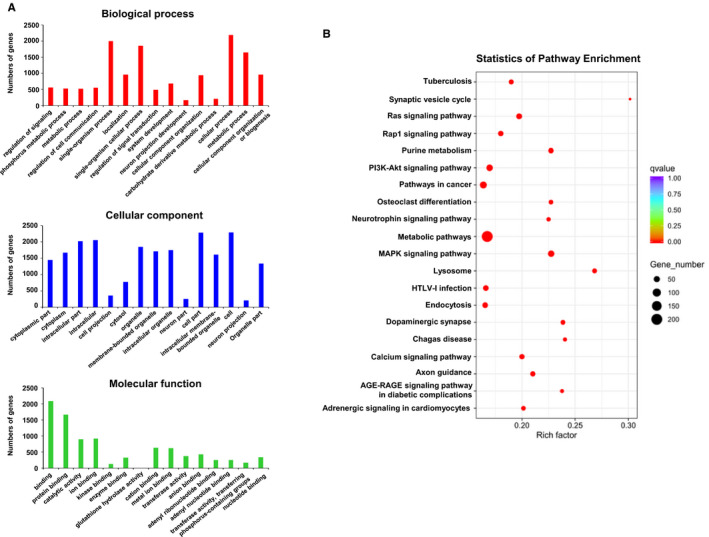
Functional enrichment analysis of differential miRNA target genes regulated by exosomal ADAMTS9‐AS2 in OSCC cells. A, GO enrichment histogram showing functions of differential miRNA target genes in biological process, cellular component, and molecular function. B, Pathway enrichment of differential miRNA target genes by exosomal ADAMTS9‐AS2 in OSCC cells. A pathway with a *P*‐value of <.05 was defined as a significantly enriched pathway

To investigate the possible functions of potential target genes of miRNAs regulated by exosomal ADAMTS9‐AS2 in OSCC cells, we performed Gene Ontology (GO) and KEGG enrichment analysis on the set of target genes by differentially expressed miRNAs between control and ADAMTS9‐AS2 exosomes groups (Table S4). GO analysis showed differentially expressed GO terms affected by ADAMTS9‐AS2 exosomes, including metabolic process, cellular component organization or biogenesis involved in biological process (Figure 7A). KEGG pathway analysis further showed significantly enriched pathways, including metabolic pathway, PI3K‐Akt signalling pathway and pathways in cancer (Figure 7B). These results indicate the potential role of exosomal ADAMTS9‐AS2 in metabolic regulation and oncogenic pathways during OSCC pathogenesis.

## 4 DISCUSSION

Oral submucosal fibrosis is a chronic, insidious oral mucosal disease related to betel nut chewing, with abnormal accumulation of collagen in the lamina propria of the oral mucosa, accompanied by epithelial atrophy or hyperplasia. OSF is mainly characterized by excessive fibrosis of the submucosal layer, which is the result of disturbed microenvironmental homeostasis between the extracellular matrix (ECM) and stromal cells such as fibroblasts, vascular endothelial cells and immune cells.^27^ Alterations in the cellular microenvironment are essential for promoting the growth and metastasis of OSF cells and have become one of the major biological mechanisms of OSF development and carcinogenesis.

Exosomal‐transferred lncRNAs regulate apoptosis, proliferation and migration of tumour cells and induce angiogenesis, with the potential to be biomarkers for tumour diagnosis and prognosis.^28^, ^29^ LncRNA ADAMTS9‐AS2 is the antisense transcript of the protein‐coding gene ADAMTS9. Here, we first identified that ADAMTS9‐AS2 was gradually down‐regulated during OSF progression, with the lowest expression levels in OSCC cells and tissues. Moreover, low ADAMTS9‐AS2 expression in OSCC was associated with poor overall survival, suggesting an important role of ADAMTS9‐AS2 during OSF progression. Recent studies have revealed that ADAMTS9‐AS2 is down‐regulated in multiple cancers including lung,^30^, ^31^ oesophageal,^32^ gastric,^33^ colon^34^ and breast,^35^, ^36^ ovarian^37^ and bladder^38^ cancers, as well as glioblastoma.^39^ Furthermore, ADAMTS9‐AS2 reduction as an important molecular marker is associated with poor survival of lung,^30^ breast,^36^ ovarian ^37^ and bladder^40^ cancers, whereas its high expression in salivary adenoid cystic carcinoma is associated with metastasis.^41^

ADATMS9‐AS2 contains a typical CGI and was frequently methylated in OSCC tissues, but rarely in OSF and normal mucous tissues, suggesting that its methylation is tumour‐specific and could be a potential early marker for OSF and OSCC detection. *ADAMTS9‐AS2* is frequently methylated in glioma and breast tumours, which may be regulated by DNMT1 and DNMT3A/B.^32^, ^42^ Moreover, *ADAMTS9‐AS* promoter methylation also could be detected in stage I breast cancer,^36^ supporting an important value of its methylation in detecting early tumours including OSCC.

Studies have demonstrated that ADAMTS9‐AS2 inhibits proliferation, cell migration and invasion, and induces apoptosis in the lung, gastric and ovarian cancer cells.^30^, ^34^, ^37^ We here found that ADAMTS9‐AS2 could be packaged into exosomes and secreted into cell medium. Exosomal ADAMTS9‐AS2 significantly suppressed malignant behaviours of OSCC cells by inhibiting cell growth, migration and invasion. The tumour‐suppressive effects exerting by exosomal ADAMTS9‐AS2 in OSCC tumorigenesis are consistent with those effects in cellular ADAMTS9‐AS2 in oesophageal, lung and breast cancers. Mechanistically, we found that exosomal ADAMTS9‐AS2 suppressed PI3K‐AKT signalling pathways and EMT in OSCC cells. ADAMTS9‐AS2 suppressed tumour spheroid formation of gastric cells through down‐regulating SPOP.^34^ Further investigation of exosomal ADAMTS9‐AS2 on stemness of OSF progression is needed.

In this study, we purified exosomes from the supernatant of OSCC cells with overexpression of ADAMTS9‐AS2 and established the miRNA expression profiles of exosomes derived from control and ADAMTS9‐AS2–expressing OSCC cells using miRNA‐seq. The low proportion of well‐known miRNA in exosomal small RNA is mainly because of the specificity of exosomal samples. The built library‐enriched RNA contains a large number of degradation fragments of other RNAs, so the classification annotation results have a high ratio of lncRNA degradation fragments (other) and mRNA degradation fragments (exon, intron) to columns; thus, the proportion of known miRNAs is only about 10% below. Through analysing target genes of miRNAs regulated by exosomal ADAMTS9‐AS2 from OSCC cells, we found several significantly enriched pathways including metabolic pathway, PI3K‐Akt signalling pathway and pathways in cancer, which further supports the roles of exosomal ADAMTS9‐AS2 in this study.

## 5 CONCLUSIONS

**FIGURE 8:**
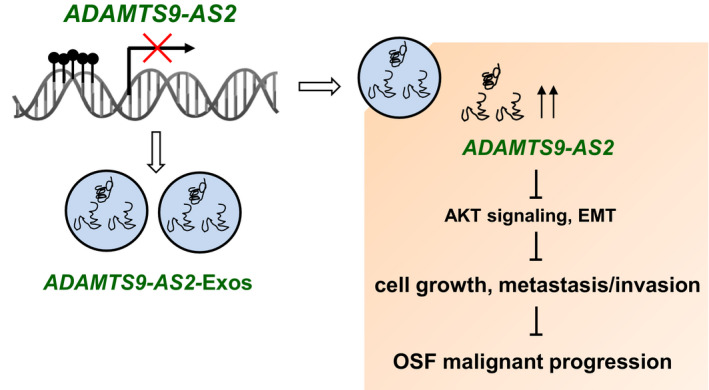
Schematic diagram of the role of exosomal ADAMTS9‐AS2 during OSF progression. Exosomal ADAMTS9‐AS2–derived OSCC tumour cells suppress cell growth, metastasis and invasion through regulating epithelial‐mesenchymal transition (EMT) and AKT signalling pathway

Thus, our results indicated that exosomal ADAMTS9‐AS2 was a promising novel biomarker for early detection of OSCC. Exosomal ADAMTS9‐AS2 could transport to the cell microenvironment and exerts tumour‐suppressive roles like exogenous ADAMTS9‐AS2. Furthermore, exosomal ADAMTS9‐AS2 suppresses OSCC progression by inhibiting AKT signalling pathway and EMT (Figure 8). Together, our results revealed that exosomal ADAMTS9‐AS2 serves as a functional mediator in cell‐cell communication, which provides further evidence of the importance of exosomal lncRNAs during OSF carcinogenesis.

## CONFLICT OF INTEREST

The authors declare no conflict of interest.

## AUTHOR CONTRIBUTIONS

**Shanghui Zhou:** Conceptualization (equal); Data curation (equal); Formal analysis (equal); Funding acquisition (equal); Writing‐original draft (equal); Writing‐review & editing (equal). **Yun Zhu:** Data curation (equal); Formal analysis (equal); Methodology (equal). **Zhenming Li:** Data curation (equal); Formal analysis (equal); Methodology (equal). **Yonggan Zhu:** Data curation (equal); Formal analysis (equal); Methodology (equal). **Zhijing He:** Methodology (equal); Resources (equal). **Chengping Zhang:** Investigation (equal).

## ETHICAL APPROVAL

This study was approved by the Institutional Review Boards of the Xiangya School of Medicine or Shanghai Jiaotong University School of Medicine and conformed to the principles of the Declaration of Helsinki.

## Supporting information

Tab S1

Tab S2

Tab S3

Tab S4

## Data Availability

RNA‐Seq data generated in this study are deposited in the Gene Expression Omnibus (GEO) database under the accession number ‘GSE125866’.
